# Association between inflammatory bowel disease and osteoporosis in European and East Asian populations: exploring causality, mediation by nutritional status, and shared genetic architecture

**DOI:** 10.3389/fimmu.2024.1425610

**Published:** 2024-07-29

**Authors:** Jian Kang, Xize Wu, Yue Li, Shuangli Zhao, Shixuan Wang, Dongdong Yu

**Affiliations:** ^1^ Graduate School, Liaoning University of Traditional Chinese Medicine, Shenyang, China; ^2^ Department of Cardiology, Affiliated Hospital of Liaoning University of Traditional Chinese Medicine, Shenyang, China; ^3^ Orthopedics and Traumatology, The Second Affiliated Hospital of Liaoning University of Traditional Chinese Medicine, Shenyang, China; ^4^ Orthopedics and Traumatology, Affiliated Hospital of Liaoning University of Traditional Chinese Medicine, Shenyang, China; ^5^ Key Laboratory of Ministry of Education for Traditional Chinese Medicine Viscera-State Theory and Applications, Liaoning University of Traditional Chinese Medicine, Shenyang, China

**Keywords:** inflammatory bowel disease, osteoporosis, genetic correlation, causality, mediating effect, multi-trait analysis, harmful variant

## Abstract

**Background:**

While previous research has established an association between inflammatory bowel disease (IBD) and osteoporosis (OP), the nature of this association in different populations remains unclear.

**Objective:**

Our study used linkage disequilibrium scores(LDSC) regression analysis and Mendelian randomization(MR) to assess the genetic correlation and causal relationship between IBD and OP in European and East Asian populations.

**Methods:**

We performed separate genetic correlation and causal analyses for IBD and OP in European and East Asian populations, used the product of coefficients method to estimate the mediating effect of nutritional status on the causal relationship, and used multi-trait analysis to explore the biological mechanisms underlying the IBD-nutrition-OP causal pathway.

**Results:**

Our analysis revealed a significant genetic correlation and causal relationship between IBD and OP in the European population. Conversely, no such correlation or causal relationship was observed in the East Asian population. Mediation analysis revealed a significant mediating effect of nutritional status on the causal pathway between IBD and OP in the European population. Multi-trait analysis of the IBD-nutrition-OP causal pathway identified MFAP2, ATP13A2, SERPINA1, FTO and VCAN as deleterious variants.

**Conclusion:**

Our findings establish a genetic correlation and causal relationship between IBD and OP in the European population, with nutritional status playing a crucial mediating role.

## 1 Introduction

Bone is a dynamic and actively remodeling tissue that maintains a delicate equilibrium between bone formation by osteoblasts and bone resorption by osteoclasts (1). OP arises from an imbalance in bone homeostasis in which bone resorption exceeds bone formation, resulting in reduced bone mass, deteriorated microarchitecture, and heightened susceptibility to fragility fractures (2). In 2010, 5.5 million men and 22 million women in the European Union were diagnosed with OP (3). The prevalence of OP is steadily increasing due to changing global demographics, earning it the moniker ‘the silent disease of the 21st century’ (4).

IBD is defined as an autoimmune disorder that primarily affects the gastrointestinal tract. Its chronic and relapsing nature makes it a challenging disease to treat. Currently, no known cure exists for IBD (5). IBD is divided into two primary subtypes, Crohn’s disease(CD) and ulcerative colitis(UC), based on the affected area within the digestive tract. CD typically affects the terminal ileum, cecum, perianal region, and adjacent colon, while UC usually involves the rectum, with continuous extension to encompass a portion of or the entire colon (6–8). Since its identification a century ago, Europe has consistently exhibited the highest incidence of IBD (9). Reports indicate that over 2.5 million individuals in Europe have received an IBD diagnosis (10). In recent years, there has been a significant change in the epidemiology of IBD, with a noticeable rise in Asian countries and among Asian immigrant populations in Western nations (11, 12). Research suggests an increased risk of OP in individuals with IBD (13–15). However, the potential variation in the link between these two conditions across European and East Asian populations remains uncertain. Therefore, it is crucial to conduct further investigation into the causal relationship and shared genetic architecture between IBD and OP within diverse ethnic groups for a comprehensive understanding of both diseases.

Nutritional status is defined as the overall condition of the body resulting from the intake, absorption and utilization of nutrients, including the effects of specific physiological and pathological states (16). Malnutrition includes deficiencies, imbalances, or excesses of energy and nutrients, including micronutrients. It has been shown that chronic bowel inflammation in IBD patients interferes with the absorption of essential micronutrients, including Fe, Ca, vitamin D, vitamin B12, folic acid, Zn, Mg and vitamin A, resulting in deficiencies (17). In addition, research has identified Ca and vitamin D as key micronutrients in mitigating bone loss, with vitamin B12, folic acid, vitamin K, vitamin C, phosphate, Mg and Na also playing a role (18–22). Previous research strongly suggests that malnutrition is a significant factor in the development of OP in people with IBD. Body composition assessment is an important tool to effectively assess and monitor nutritional status, with bioelectrical impedance analysis emerging as the most efficient and reliable method due to its rapid performance, non-invasive nature and cost-effectiveness (23, 24). Therefore, we will use impedance of arm(IOA) as an indicator of nutritional status to investigate the mediating effect and common genetic architecture of nutritional status within the causal pathway linking IBD and OP, with the aim of gaining new insights into how IBD influences the development of OP.

## 2 Methods

### 2.1 Research design

**Figure 1:**
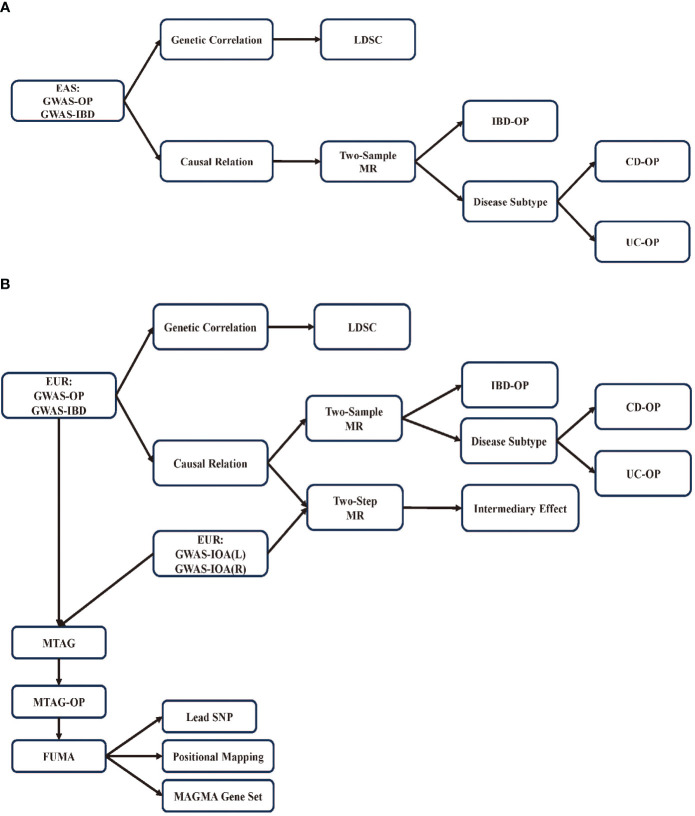
Research flowchart. **(A)** Flowchart for research on East Asian population; **(B)** Flowchart for research on European population.

This study examines the genetic correlation and causal relationships between IBD and OP in European and East Asian populations. To evaluate the genetic correlation between IBD and OP, we used LDSC. We conducted two-sample MR analyses to assess the causal relationship between IBD and OP. We then employed a two-step approach to evaluate the mediating effect of IOA. Finally, we performed multi-trait analysis using MTAG to investigate the shared genetic architecture underlying IBD, IOA, and OP (**Figure 1**).

### 2.2 Data source

To identify genetic variants associated with IBD, data from the International Inflammatory Bowel Disease Genetics Consortium (IIBDGC, https://www.ibdgenetics.org/) was used. The IIBDGC conducted the largest multi-ancestry (East Asian and European) IBD genetics study to date (25). The analysis of East Asian population included 14,393 cases of IBD, consisting of 7,372 cases of CD and 6,862 cases of UC, as well as 15,456 controls. Samples were collected from East Asian countries such as China, South Korea, and Japan. The analysis of European population involved 25,042 cases of IBD and 34,915 controls of non-Finnish European population. This included 12,194 cases of CD and 28,072 controls, as well as 12,366 cases of UC and 33,609 controls. The original GWAS researchers conducted thorough quality control and association analyses to effectively control for population structure and other confounding factors.

The GWAS summary dataset for investigating the genetic associations of OP in individuals of European population, including 8,017 cases and 391,037 controls, was provided by the FinnGen biobank (https://r10.finngen.fi/pheno/M13_OSTEOPOROSI) (26). A summary dataset of GWAS was obtained to explore the genetic associations of OP in individuals of East Asian population. The dataset comprised data from 9,794 cases and 168,932 controls of Japanese descent (https://pheweb.jp/) (27). OP diagnoses in this study were ascertained from hospital statistics and categorized using the International Classification of Diseases and Related Health Problems (ICD) 10 coding system.

The GWAS summary datasets investigating the genetic associations of IOA were sourced from the MRC-IEU database (https://gwas.mrcieu.ac.uk/). The dataset was generated using the Phesant GWAS pipeline on derived variables from UKBiobank. It included 454,850 individuals of European population for left IOA and 454,862 individuals of European population for right IOA (28).

### 2.3 Genetic correlation

LDSC is a commonly used method for analyzing genetic correlations. It allows for the estimation of genetic contributions to complex diseases and traits by utilizing the concept of LD. In this study, we used the 1000 Genomes Project as the reference panel to calculate LD scores, following established methodology. The strength of association between individual single nucleotide polymorphisms (SNPs) and complex traits was inferred by estimating their respective LD Scores (29). LDSC was used to conduct cross-trait analysis and assess the genetic correlation between IBD and OP.

### 2.4 Causal relation

#### 2.4.1 Two-sample MR

Valid results from MR analysis depend on the fulfillment of three key assumptions. Specifically, genetic variants that serve as instrumental variables(IVs) for risk factors must satisfy the following: (1) relevance: showing a robust association with the risk factor under study; (2) independence: showing no correlation with known or unknown confounders; and (3) exclusion restriction: influencing the outcome solely through the risk factor, with no other direct causal pathways (30). To ensure the relevance and exclusion restriction assumptions, we set a genome-wide significance threshold of p*<*5×10^−8^ to select exposure-associated SNPs, and a threshold of R^2^< 0.001 to identify independent variants and mitigate bias due to LD. We calculated the F statistic to minimize weak instrument bias and to assess the strength of the selected SNPs, setting a threshold of F statistic*>*10 for further analysis (31). To ensure objectivity, we used the Steiger-Flering method for SNP screening (32). In addition, we used the Mendelian Randomization Pleiotropy RESidual Sum and Outlier (MR-PRESSO) test to identify potential outliers within the multi-instrument MR analysis, particularly those with pleiotropic effects at the SNP level (33). Based on previous research, we identified age at menopause and age at menarche as potential confounders of the independence assumption. We then used the LDlink database (https://ldlink.nih.gov/) to query traits associated with IVs (R^2^ = 0.8, base pair window=50000) and subsequently removed SNPs associated with these confounders (34). Inverse variance weighted (IVW) analysis served as the primary MR analysis, with the weighted median method, MR-Egger, and MR-PRESSO used to assess the robustness of the IVW results (33, 35, 36). We used the Q test for both IVW and MR-Egger to identify heterogeneity among individual IVs that could potentially violate the assumptions. MR-Egger, through its intercept estimate, was used to assess pleiotropy and ensure the independence of genetic variation from the outcome (37). In addition, we conducted MR analyses stratified by European and East Asian populations and further by disease subtypes (CD and UC) to investigate the causal relationship between IBD subtypes and OP across populations.

#### 2.4.2 Two-step MR

IVW method was used as the primary approach to estimate the causal effects in the two-step MR mediation analysis. This analysis involved the calculation of two different MR effects: (1) the causal effect of exposure on the mediator ( $\hat{a}$ ); the causal effect of the mediator on the outcome ( $\hat{b}$ ). The product of coefficients method was used as the primary approach to estimate the indirect effect, specifically the mediating influence of IBD on OP through IOA (38).

### 2.5 Multi-trait analysis of GWAS

MTAG is a meta-analytical approach that uses inter-trait correlations to increase statistical power for detecting new genetic associations within individual traits. This method applies inverse-variance weighted meta-analysis to summary statistics obtained from GWAS conducted on diverse traits (39). MTAG assumes the homogeneity of the effect-size variance-covariance matrix across SNPs for all traits. Even in cases where this assumption is not met, for instance, when specific SNPs influence only a subset of traits, the estimators derived from MTAG can maintain consistency.

FUMA was used to functionally map and annotate significant SNPs identified through GWAS analysis using MTAG. The aim was to elucidate the genetic underpinnings linking OP, IBD, and arm resistance (40). SNP2GENE was used to annotate the biological functions of SNPs and their corresponding gene mapping. The parameters for independent significant SNPs and lead SNPs were set as follows: a maximum P-value of p<5×10^−8^, a maximum P-value cutoff of 0.05, an r^2^ threshold of ≥ 0.6, a secondary r^2^ threshold of ≥ 0.1, and a maximum distance of< 250 kb between LD blocks for merging into a single locus.

### 2.6 Statistical analysis

LDSC (v1.0.0, https://github.com/bulik/ldsc). R software (version 4.3.2), and R packages ‘TwoSampleMR’, ‘MendelianRandomization’, ‘MRPRESSO’, and ‘MVMR’ were used for all MR analyses. MTAG (0.9.0, https://github.com/JonJala/mtag) and FUMA (https://fuma.ctglab.nl/) were used for data cleaning and statistical/bioinformatics analysis.

## 3 Results

### 3.1 Genetic correlation

Single trait LDSC regression results: The heritability (h2) of OP in individuals of European population was estimated to be 0.0102 (se:0.0017), with mean Chi^2^ statistic of 1.1247 and intercept of 1.0442 (se:0.0084). The h2 of IBD was estimated to be 0.3109 (se: 0.0302), with mean Chi^2^ statistic of 1.5014 and intercept of 1.1298 (se:0.0144). In individuals of East Asian population, the h2 of OP was estimated at 0.0139 (se: 0.0028),with mean Chi^2^ statistic of 1.1247 and intercept of 1.0442 (se:0.0084).The h2 of IBD was estimated at 0.3146 (se: 0.0357). with mean Chi^2^ statistic of 1.2398 and intercept of 1.0478 (se:0.01).The results of the Cross-Trait LD Score Regression Genetic Correlation Analysis are as follows: (1) For individuals of European population, the genetic correlation (rg) between OP and IBD was estimated at 0.1278 (se:0.0577), with Z-score of 2.2139 and p-value of 0.0268. (2) For individuals of East Asian population, the genetic correlation between OP and IBD was estimated at 0.0696 (se: 0.086), with Z-score of 0.8095 and p-value of 0.4182.

### 3.2 Causal relation

#### 3.2.1 Two-sample MR

**Figure 2:**
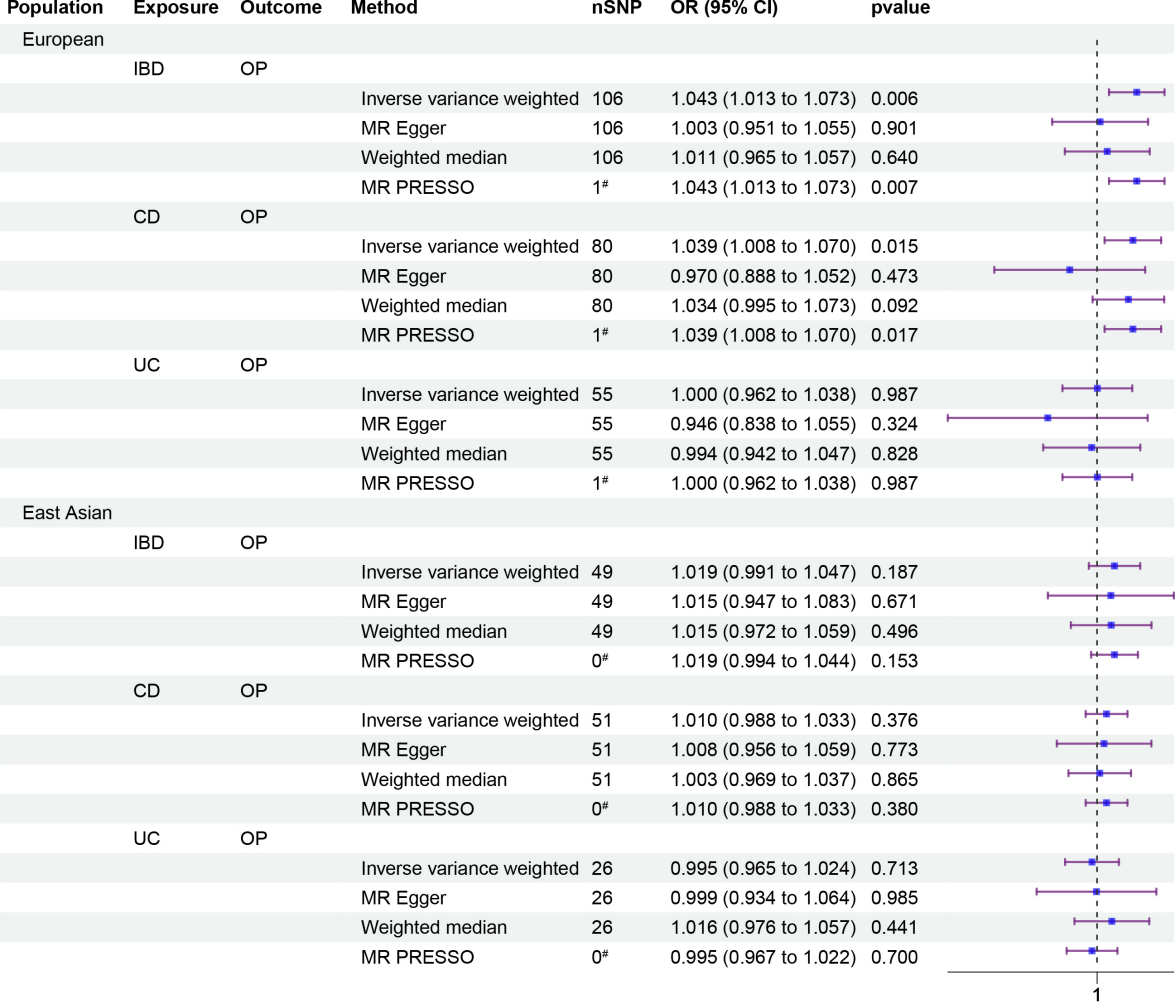
Forest plots of causality. Forest plots including causality between IBD and OP of East Asian population and European population in the analyses and causality between disease subtypes CD, UC and OP. The forest plot includes nSNP, Beta95%CI, and correlation pvalues for all studies in the analysis. ^#^ represents the number of outliers filtered by MR-PRESSO.

To investigate the causal relationship between IBD and OP, we performed a univariate Mendelian randomization (UVMR) analysis and identified suitable SNPs as IVs using F-statistics, Steiger filtering, and outlier diagnostics. Interestingly, we found a significant positive causal relationship between IBD and OP in the European population (odds ratio [OR]: 1.043, 95% CI: 1.013–1.073, p = 0.006), but this association was not present in the East Asian population. Further analysis of disease subtypes showed a positive causal relationship between CD and OP in the European population (OR: 1.039, 95% CI: 1.008–1.07, p = 0.015), but not between UC and OP. In the East Asian population, no causal relationships were observed between either CD or UC and OP (**Figure 2**).

#### 3.2.2 Two-step MR

**Figure 3:**
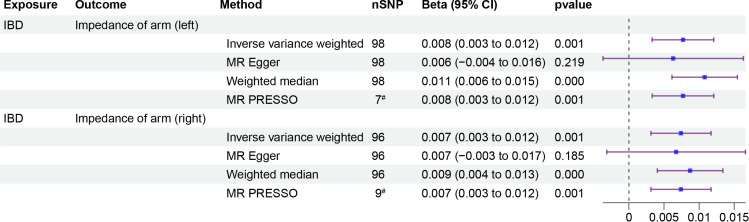
Two-step MR analysis of forest plots. Forest diagrams include analyzing the causal relationship between IBD and IOA. The forest plot includes nSNP, Beta95%CI, and correlation pvalues for all studies in the analysis. ^#^ represents the number of outliers filtered by MR-PRESSO.

**Figure 4:**
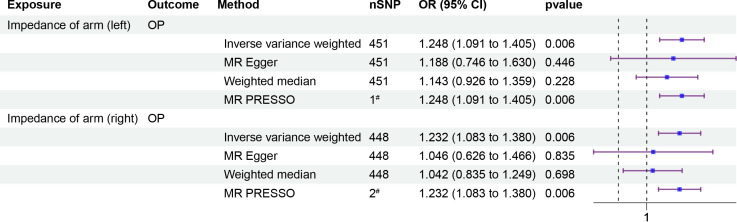
Two-step MR analysis of forest plots. Forest diagrams include analyzing the causal relationship between IOA and OP. The forest plot includes nSNP, Beta95%CI, and correlation pvalues for all studies in the analysis. ^#^ represents the number of outliers filtered by MR-PRESSO.

To investigate whether IOA mediates the causal relationship between IBD and OP, we analyzed individuals of European population who showed a causal link between IBD and OP. Our causal analysis revealed a positive association between IBD and IOA, with *β* of 0.008 (95% CI: 0.003, 0.012, p = 0.001) for the left arm and 0.007 (95% CI: 0.003, 0.012, p = 0.001) for the right arm (**Figure 3**). Further analysis showed a positive association between IOA and OP, OR were 1.248 (95% CI: 1.091, 1.405, p = 0.006) for the left arm and 1.232 (95% CI: 1.083, 1.380, p = 0.006) for the right arm (**Figure 4**). We estimated the mediating effect of IOA on the IBD-OP causal pathway using the coefficient product method, OR were 1.002 (95% CI: 1.000, 1.004) for the left arm and 1.001 (95% CI: 1.000, 1.003) for the right arm.

### 3.3 Multi-trait analysis of GWAS

**Figure 5:**
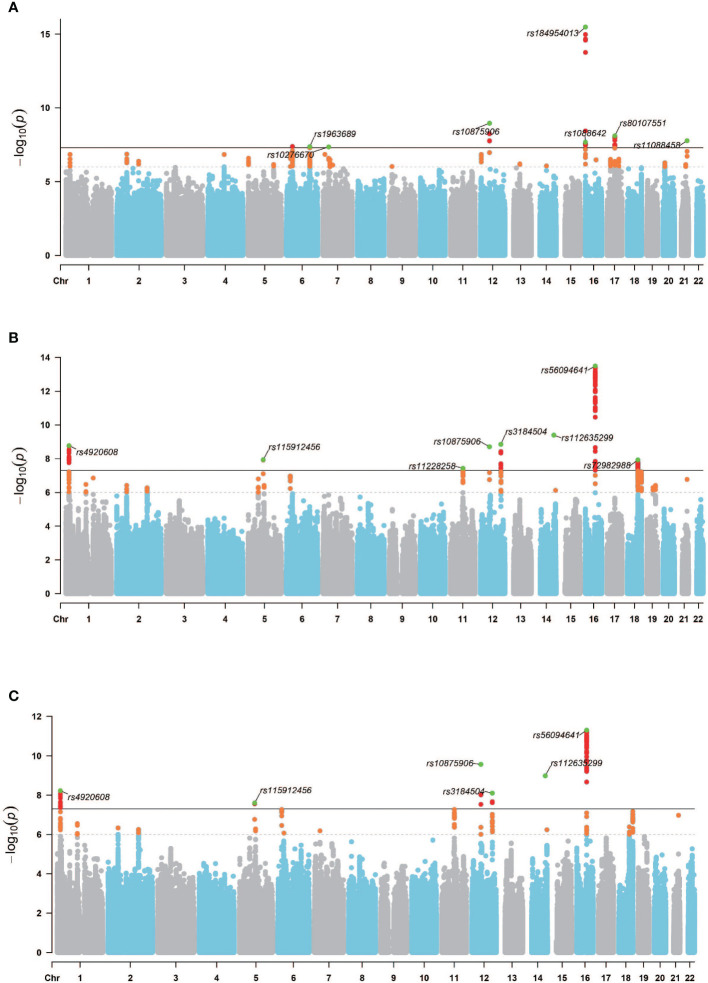
GWAS Manhattan chart. **(A)** *GWAS* − *OP_original_* Manhattan map, **(B)** *MTAG* − *OP_IBD−IOA_*_(_ *_L_*_)_ Manhattan map, **(C)** *MTAG* − *OP_IBD−IOA_*_(_ *_R_*_)_ Manhattan map. Horizontal coordinates are chromosomes and vertical coordinates are -*log*_10_(p). The dashed line indicates p threshold of 1× 10^−6^, and the solid line indicates p threshold of 5× 10^−8^. Orange dots indicate SNPs 1× 10^−6^ *<*p*<*5× 10^−8^, red dots indicate p*<*5× 10^−8^, and green dots indicate lead SNPs.

Considering the established genetic correlation and causal link between OP and IBD in European population, along with the mediating role of IOA in this relationship, we performed a multi-trait GWAS analysis. This analysis utilized GWAS data for OP, IBD, and IOA (both left and right) to identify genetic loci within the OP GWAS dataset that exhibit associations with IBD and IOA. SNPs reaching genome-wide significance and clustered based on an R^2^ threshold of 0.1 to ensure approximate independence were designated as “lead SNPs”. Following MTAG integrating GWAS data for OP with both GWAS-IBD and GWAS-IOA, the initial set of 50 SNPs exhibiting significant associations (p*<*5×10^−8^) in the original GWAS-OP expanded to encompass 146 SNPs for the left IOA and 94 SNPs for the right IOA within the MTAG-OP results. As defined previously, “lead SNPs” represent SNPs achieving genome-wide significance and clustered with an R^2^ value of 0.1 to approximate independence. The original GWAS-OP dataset contained 7 lead SNPs. Following MTAG analysis, the MTAG-OP results revealed 8 lead SNPs for the left IOA and 6 lead SNPs for the right IOA that demonstrated associations with both IBD and IOA (**Figure 5**). The analysis of the genomic loci identified 25 genes for the left IOA and 23 genes for the right IOA exhibiting associations with the IBD-IOA-OP relationship. Among these, 6 genes for the left IOA and 5 genes for the right IOA harbored deleterious variants with a Combined Annotation Dependent Depletion (CADD) score exceeding 12.37. By integrating the MTAG analysis results for both left and right IOAs in the context of the IBD-IOA-OP relationship, we identified five genes harboring deleterious variants: MFAP2, ATP13A2, SERPINA1, FTO, and VCAN. We performed MAGMA gene set analysis using curated gene sets and Gene Ontology (GO) terms sourced from the Molecular Signatures Database (MsigDB). Integrating the results of the MTAG analysis results for both IOAs in relation to the IBD-IOA-OP association, we identified four significant gene sets: GOBP SKELETAL SYSTEM DEVELOPMENT, GOBP_REGULATION_OF_SKELETAL_MUSCLE_CELL_DIFFERENTIATION, WP_ENDOCHONDRAL_OSSIFICA and WP_ENDOCHONDRAL_OSSIFICATION_WITH_SKELETAL_DYSPLASIAS.

## 4 Discussion

To our knowledge, this study pioneers a comprehensive cross-trait analysis of the IBD-IOA-OP causal pathway using whole-genome data, providing a systematic assessment of their shared genetic architecture based on balanced evidence for both genetic correlation and causation. Initially, we employed LDSC and MR analyses to investigate the genetic correlation and causality between IBD and OP across European and East Asian population. Our findings revealed a positive genetic correlation (rg: 0.1278) and causal relationship (OR: 1.043, 95%CI (1.013, 1.073)) between IBD and OP within the European population. However, this correlation and causality were absent in the East Asian population. Subsequently, disease subtype analysis revealed a positive causal relationship between CD and OP (OR: 1.039, 95%CI (1.008, 1.070)) within the European population. Conversely, no causal relationship was observed between UC and OP in either European or East Asian population, nor between CD and OP in East Asian population. Finally, a two-step MR analysis identified the mediating effects of IOA on the causal pathway of IBD to OP within European population, specifically for the effects of the left side (OR: 1.002, 95% CI (1.000, 1.004)) and right side (OR: 1.001, 95% CI (1.000, 1.003)). Using MTAG for multi-trait causal pathway analysis, we identified deleterious variants MFAP2, ATP13A2, SERPINA1, FTO, and VCAN within the causal pathway IBD-IOA-OP. In conclusion, our research provides compelling evidence for genetic correlation and causality between IBD and OP in European population, elucidates the IBD-IOA-OP causal pathway and common genetic basis, and identifies potential functional genes associated with this pathway. It is important to acknowledge the diverse genetic backgrounds across different ethnicities, which may explain the lack of robust genetic evidence supporting the correlation and causality between IBD and osteoporosis in East Asian population.

Osteopenia and osteoporosis are prevalent extraintestinal manifestations in IBD patients, affecting 40–50% and 2–30% of patients, respectively (41, 42). Epidemiological studies within European population have revealed a higher prevalence of reduced bone mineral density(BMD) in IBD patients. There is evidence that IBD patients exhibit reduction in BMD, with CD patients exhibiting more pronounced reduction compared to UC patients, who demonstrate either no significant reduction or less pronounced decrease (43–45). MR analysis conducted on European population identified positive causal relationship between IBD and OP. Subtype analysis revealed positive causal association between CD and OP, while no such association was observed for UC (46). Our findings corroborate these results, suggesting that the divergent causal relationships between CD and UC with OP may be attributed to malnutrition resulting from impaired absorption (47). The increased propensity for malnutrition in CD compared to UC may be attributed to its location in the small intestine and its nature as a systemic disease with a prolonged pre-disease period. In contrast, UC is confined to the distal colon and presents as an acute-onset mucosal disease (48). Epidemiological studies investigating the association between IBD and OP within East Asian population remain limited, with conflicting findings. A study focusing on postmenopausal women in China identified IBD as a non-significant risk factor for OP (49). A comprehensive Korean study examining the prevalence of extraintestinal manifestations in IBD patients revealed a higher prevalence of osteoporosis within this group. However, the standardized prevalence ratio compared to the general Korean population did not indicate a significant association, suggesting that IBD may not be a primary factor influencing OP development (50). Conversely, other studies have identified IBD as a significant risk factor for OP (51, 52). Our findings align with previous epidemiological studies and MR analyses conducted within European population. However, the results observed in our East Asian population diverge from both European data and some prior East Asian epidemiological and MR analyses. Several factors may contribute to these discrepancies: (1) Population genetic structure contributes to differences in osteoporosis-associated genetic variants between European and East Asian populations. GWAS were conducted separately for European and East Asian populations to identify genetic variants associated with osteoporosis. In contrast to the European population, no significantly associated loci, independent SNPs, and lead SNPs were identified for osteoporosis phenotypes in the East Asian population under the same statistical threshold. The European population revealed 6 loci, 9 independent SNPs, and 7 lead SNPs significantly associated with osteoporosis phenotypes[(see the Manhattan plots comparing GWAS results for osteoporosis in the two populations in **Supplementary Figures S7**, **S8**)]. The findings indicate that diverse mechanisms influencing the distribution and function of genetic variants across populations contribute to the substantial differences in osteoporosis-associated genetic variants between European and East Asian populations. (2) While rapid urbanization and industrialization in East Asian countries (China, South Korea, and Japan) have resulted in an exponential rise in IBD prevalence, rates remain considerably lower than those observed in European nations (53, 54). Additionally, variations in industrial development and healthcare systems across East Asia contribute to regional disparities in IBD prevalence and extraintestinal manifestations (55). (3) The emergence of IBD in previously unaffected regions typically follows a pattern, with UC initially presenting more frequently than CD. Geographic variation in the distribution of IBD subtypes has been observed, with UC being predominant in East Asian countries and CD being more prevalent in European nations. The anatomical location of inflammation in UC may explain the lower incidence of malnutrition-induced osteoporosis observed in these patients (56). The reported prevalence ratio of UC to CD in Asia is 2.0, further highlighting the regional differences in IBD subtype distribution (57). (4) Other confounding factors also contribute to the observed disparities. Dietary habits, such as the prevalent consumption of tea in East Asian countries and coffee in European countries, may play a role. It has been suggested that coffee consumption may exacerbate gastrointestinal symptoms in IBD patients, leading to decreased intestinal Ca absorption and disruption of Ca-P homeostasis, which may ultimately contribute to osteoporosis development (58, 59). Conversely, tea consumption has been linked to reduced intestinal inflammation and permeability, potentially offering protection against IBD progression. Furthermore, tea consumption has been associated with increased bone-specific alkaline phosphatase levels, a marker of bone formation, which may contribute to a reduction in the risk of osteoporosis (60–62).

This study makes significant contribution by exploring the novel domain of IBD-nutrition-OP interactions. We have identified and quantified the mediating role of the index of overall nutritional status (IOA) in the causal relationship between IBD and OP. While direct investigations into this specific pathway are limited, existing research provides supporting evidence. Bioelectrical impedance analysis has emerged as a valuable tool for accurately and efficiently assessing nutritional status and body composition in IBD patients (63–65). Moreover, research has established robust correlation between bioelectrical impedance measurements and osteoporosis (66, 67). Based on existing research, we hypothesize that the mechanism underlying the IBD-nutrition-OP causal pathway may involve two key aspects. Firstly, IBD is characterized by gut microbiota dysbiosis, which results in an imbalance between beneficial and harmful bacterial taxa and an abnormal abundance and diversity of the gut microbiota (68–71). This dysbiosis impacts the absorption, bioavailability, and bioaccessibility of essential micronutrients relevant to bone metabolism, such as Ca, Zn, Se, and Fe. Studies have demonstrated that the gut microbiota can enhance Ca absorption and regulate intestinal serotonin production, a neurotransmitter believed to interact with osteoblasts and regulate bone metabolism (72). Furthermore, research has shown that Lactobacillus plantarum 299v significantly increases the absorption of nonheme dietary iron during specific testing periods (73). Additionally, Zn and Fe Se compounds present in food sources are converted to selenomethionine (SeMet) by the gut microbiota, which enables the host to absorb this essential micronutrient. These compounds are not absorbed by the small intestine and reach the colon, where commensal bacteria enhance their bioavailability and deliver them to the host (74, 75). Secondly, IBD is a chronic, progressive inflammation within the gut that causes damage to the intestinal structure and function. This results in a wide range of gastrointestinal symptoms. IBD patients experience alterations in the intestinal mucosa, damage to the digestive tract, impaired digestion, exacerbated inflammatory consumption, hindered nutrient absorption, leading to malnutrition and subsequent significant changes in body composition. These changes are characterized by marked reductions in muscle mass and skeletal muscle mass, ultimately increasing the risk of osteoporosis (76–81).

It is notable that our research extended the analysis of the IBD-nutrition-OP causal pathway, identifying lead SNPs (rs4920608, rs115912456, rs10875906, rs3184504, rs112635299, and rs56094641) and genes associated with potentially deleterious mutations (MFAP2, ATP13A2, SERPINA1, FTO, and VCAN). MFAP2, a component of the extracellular matrix, was the first identified protein playing a crucial role in regulating growth factor signal transduction (82). Gene expression profiling revealed the specific expression of MFAP2 within osteoblast-like cells (83). Animal models demonstrated that MFAP2-deficient mice experience progressive bone loss, coupled with elevated expression of osteoclasts and NF-*κ*B ligand receptor activator (84, 85). MFAP2 plays a pivotal role in the development of functional vasculature, which is essential for the absorption, transport, and homeostasis of nutrients within the body (82, 86). ATP13A2, a lysosomal type 5 P-type ATPase involved in polyamine transport, is associated with various neurodegenerative disorders, including autosomal recessive hereditary familial Parkinson’s disease (87, 88). The rs4920608 variant, an intronic variation located at the end of the ATP13A2 gene, may influence the polymorphism of ATP13A2.Studies have demonstrated the involvement of ATP13A2 in elemental metabolism, including Mg toxicity, Zn homeostasis, and Fe deposition (89–94). Mg, Fe, and Zn are essential for bone mineralization and metabolism. Zn, in particular, exhibits a strong association with osteoporosis, playing a pivotal role in skeletal development and bone mass maintenance (95–98). SERPINA1, which has been extensively researched, exerts a significant influence on human health. The SERPINA1 gene encodes the secreted *α*1-antitrypsin (AAT) subtype, which performs essential functions by inhibiting proteases and modulating immune responses (99). Research aimed at elucidating the novel immunomodulatory roles of active vitamin D (1,25(OH)2D3) in human CD4 T cells has revealed its ability to modulate these functions through the effective induction of *α*1-antitrypsin gene (SERPINA1) expression (100, 101). It has been demonstrated that the indirect consequences of altered immune status can result in the persistent destruction of bone tissue. It has been demonstrated that immune cells interact with osteoblasts and osteoclasts through direct cell-to-cell contact. However, it is more likely that this occurs via paracrine mechanisms, particularly in the context of a chronic low-grade inflammatory phenotype (102). Genetic research has identified a correlation between the rs112635299 variant of SERPINA1 and the levels of glycoprotein acetylated and phenylalanine (103). Glycoprotein acetylated and phenylalanine have been identified as inflammatory markers that are superior indicators of chronic low-grade inflammation compared to C-reactive protein (104). The correlation between phenylalanine and inflammatory markers suggests a connection with chronic low-grade inflammation and immune activation (105). A genetic association study identified SERPINA1 as a shared genomic region for C-reactive protein and osteoporosis (106). Furthermore, SERPINA1 gene therapy has been explored and evaluated in animal models of osteoporosis (107). FTO, a key gene in gene-diet interactions, exhibits its highest expression in the brain. Its role in amino acid sensing allows it to influence the intake of both macronutrients and micronutrients (108–110). The rs56094641 variant, an intronic variation within the FTO gene, has been demonstrated to influence FTO polymorphism during osteoblast differentiation, resulting in alterations in the expression of the osteoblast biomarkers ALPL and OPN (111). Moreover, research has demonstrated downregulation of FTO mRNA in osteoporosis patients. FTO overexpression induces osteogenic differentiation of C3H10T1/2 cells, while the GDF11-FTO-PPAR*γ* axis suppresses bone formation and promotes adipogenic differentiation of osteoporotic bone marrow mesenchymal stem cells during osteoporosis. These findings underscore the pivotal role of FTO in osteoblast demise and differentiation (112–114). The VCAN gene encodes chondroitin sulfate proteoglycan, a principal component of the extracellular matrix (115). rs115912456 is an intronic variant of the VCAN gene. The functional consequences of this variant remain undefined at present. VCAN expression was observed to exhibit a two-fold upregulation during osteoblast metabolism and an eight-fold increase during the process of altered development and regeneration of dystrophic myofibers resulting from chronic calcium influx in dystrophin-deficient myofibers (116–118). Although the genes associated with the genetic variants rs10875906 and rs3184504 do not meet the CADD threshold for identifying potentially harmful mutations, these lead SNPs demonstrate a significant association with the IBD-nutrition-OP axis. rs10875906, an intergenic variant, exhibits a significant association with bone density phenotype, although the functional implications of this association remain unclear (119). rs3184504, a missense variant in the SH2B3 gene, is linked to the inflammatory metabolite kynurenine. Plasma kynurenine has been identified as a potential biomarker for both acute and chronic inflammation involving the SH2B3 pathway, and exhibits a correlation with the inflammatory marker C-reactive protein (120).

This study elucidates the genetic correlation and causal relationship between IBD and OP in both European and East Asian populations, further establishing the mediating role of nutritional status within this causal pathway. Our research boasts several strengths. Firstly, in contrast to existing East Asian IBD GWAS (with 157,116 SNPs) and OP MR studies (121), we utilized the latest and largest East Asian IBD GWAS dataset (comprising 12,869,831 SNPs), minimizing the risk of false-positive results. Secondly, we performed cross-validation of genetic correlation and MR analyses across diverse populations, ensuring robust and reliable results. Thirdly, MR analysis incorporated rigorous IV selection through MR-PRESSO and Steiger filtering tests, accounting for potential pleiotropic effects. The identification of any outliers did not impact the causal effects observed in the initial IVW analysis. Fourthly, we employed IOA as a measure of body composition and nutritional status in our causal pathway mediation analysis, guaranteeing the credibility and rationale of the model employed to elucidate the mediating effects. Fifthly, we employed multi-trait analysis of GWAS to investigate the IBD-nutrition-OP causal pathway, marking the first exploration of this domain. Finally, we analyzed the lead SNPs and their corresponding genes within the IBD-nutrition-OP causal pathway, establishing the biological mechanisms underlying this relationship.

It must be acknowledged that the limitations of our research are considerable. Firstly, our analysis of the genetic correlation and causal relationship between IBD and OP was conducted exclusively on European and East Asian populations. While our findings indicate that this relationship exists solely within the European population, the evolving epidemiology of East Asian population and the uneven development across East Asian countries and regions may introduce bias into the results. Secondly, our chosen GWAS datasets lacked gender stratification, potentially introducing outcome bias. Thirdly, despite the exclusion of SNPs associated with confounding factors such as age at menarche and menopause in the MR analysis, the potential influence of other confounders cannot be entirely ruled out. Fourthly, the overlap in sample sources within East Asian datasets may influence the stability of our findings. Finally, while we have identified genes associated with the IBD-nutrition-OP causal pathway, further longitudinal studies and experimental investigations are crucial to comprehensively unravel the underlying biological mechanisms.

## 5 Conclusions

This study provides a comprehensive understanding of the genetic correlation and causal relationship between IBD and OP, identifies the causal mediating role of nutritional status in the IBD-OP relationship, and uses multi-trait analysis to investigate the underlying biological mechanisms of the IBD-nutrition-OP causal pathway. The results of this study provide causal evidence for the pathogenesis of bone changes in IBD patients, which may facilitate early prevention and diagnosis of bone loss in this population.

## Data availability statement

The original contributions presented in the study are included in the article/**Supplementary Material**. Further inquiries can be directed to the corresponding author.

## Author contributions

JK: Conceptualization, Data curation, Formal analysis, Investigation, Methodology, Project administration, Resources, Software, Validation, Visualization, Writing – original draft. XW: Conceptualization, Investigation, Methodology, Resources, Software, Validation, Visualization, Writing – review & editing. YL: Data curation, Formal analysis, Methodology, Supervision, Validation, Writing – review & editing. SZ: Data curation, Formal analysis, Methodology, Project administration, Resources, Supervision, Validation, Writing – review & editing. SW: Conceptualization, Funding acquisition, Methodology, Project administration, Resources, Validation, Writing – review & editing. DY: Formal analysis, Funding acquisition, Methodology, Project administration, Resources, Supervision, Validation, Writing – review & editing.
