# Supplementary material for: Association between inflammatory bowel disease and osteoporosis in European and East Asian populations: exploring causality, mediation by nutritional status, and shared genetic architecture
Source: Front Immunol. 2024 Jul 29;15:1425610. doi: 10.3389/fimmu.2024.1425610 (PMC11317921; doi:10.3389/fimmu.2024.1425610)
Supplement: Supplementary file 1 [file DataSheet_1.pdf]

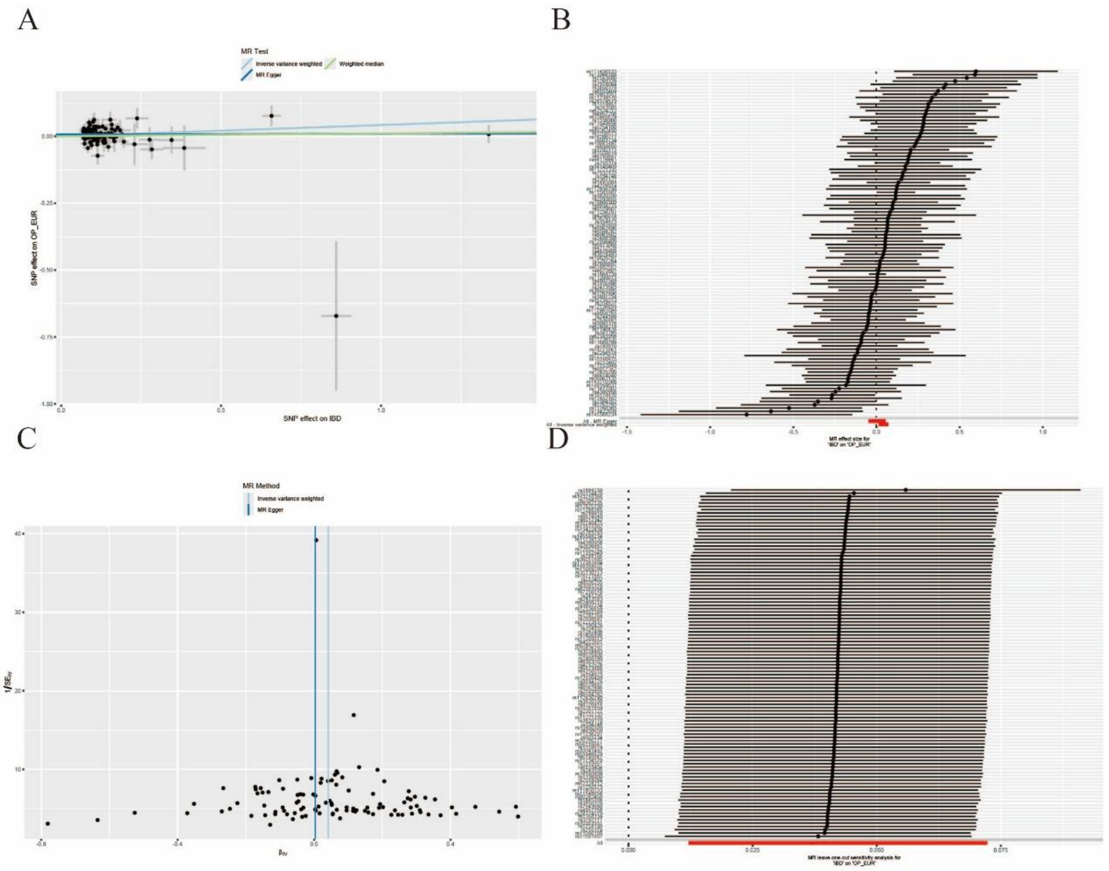

Figure S1 Scatterplot,funnel plot and leave-one-out analysis of relationship between IBD and OP in European population.

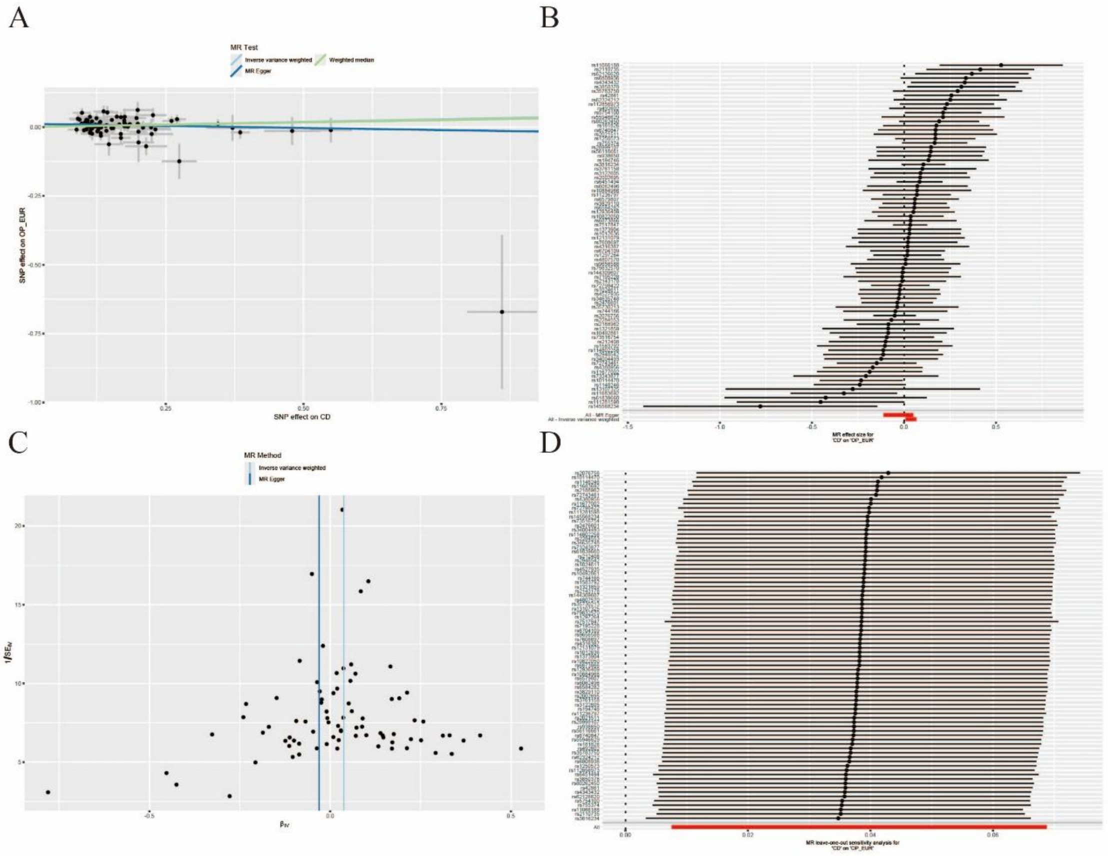

Figure S2 Scatterplot,funnel plot and leave-one-out analysis of relationship between CD and OP in European population.

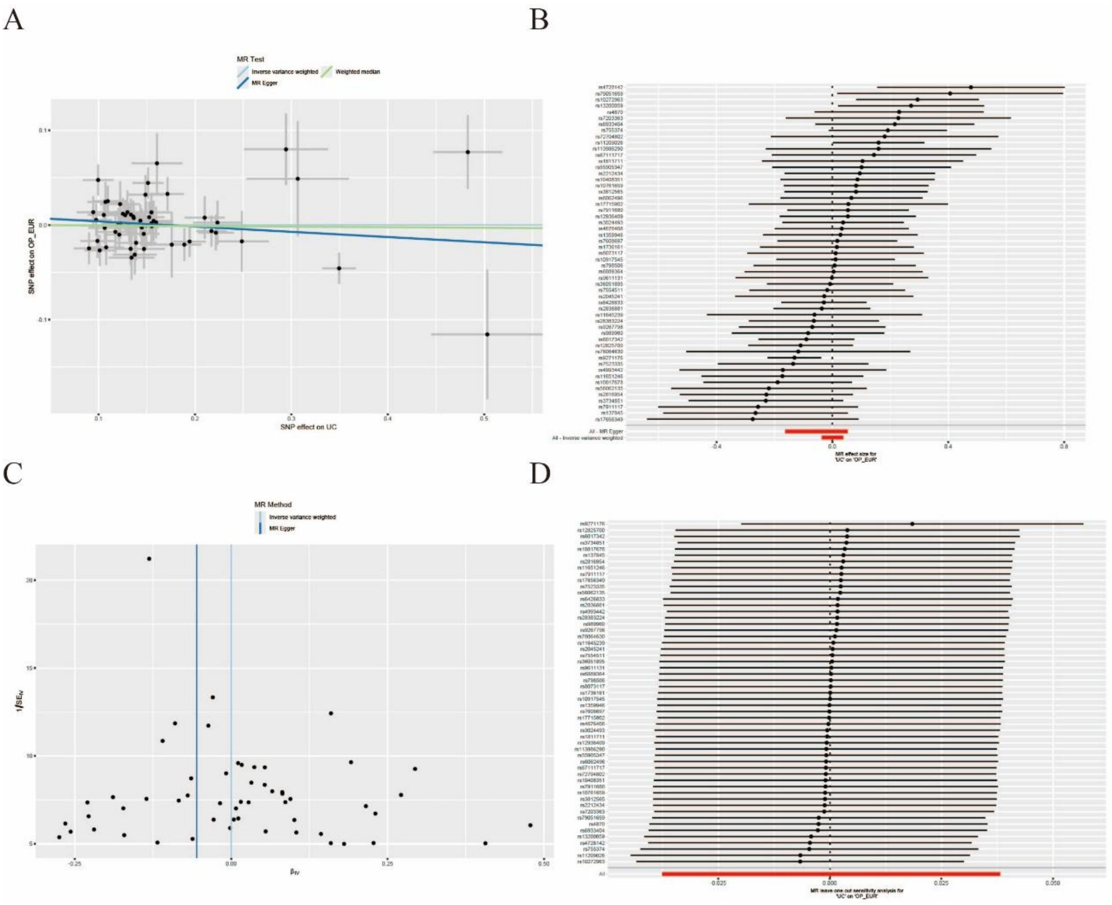

Figure S3 Scatterplot,funnel plot and leave-one-out analysis of relationship between UC and OP in European population.

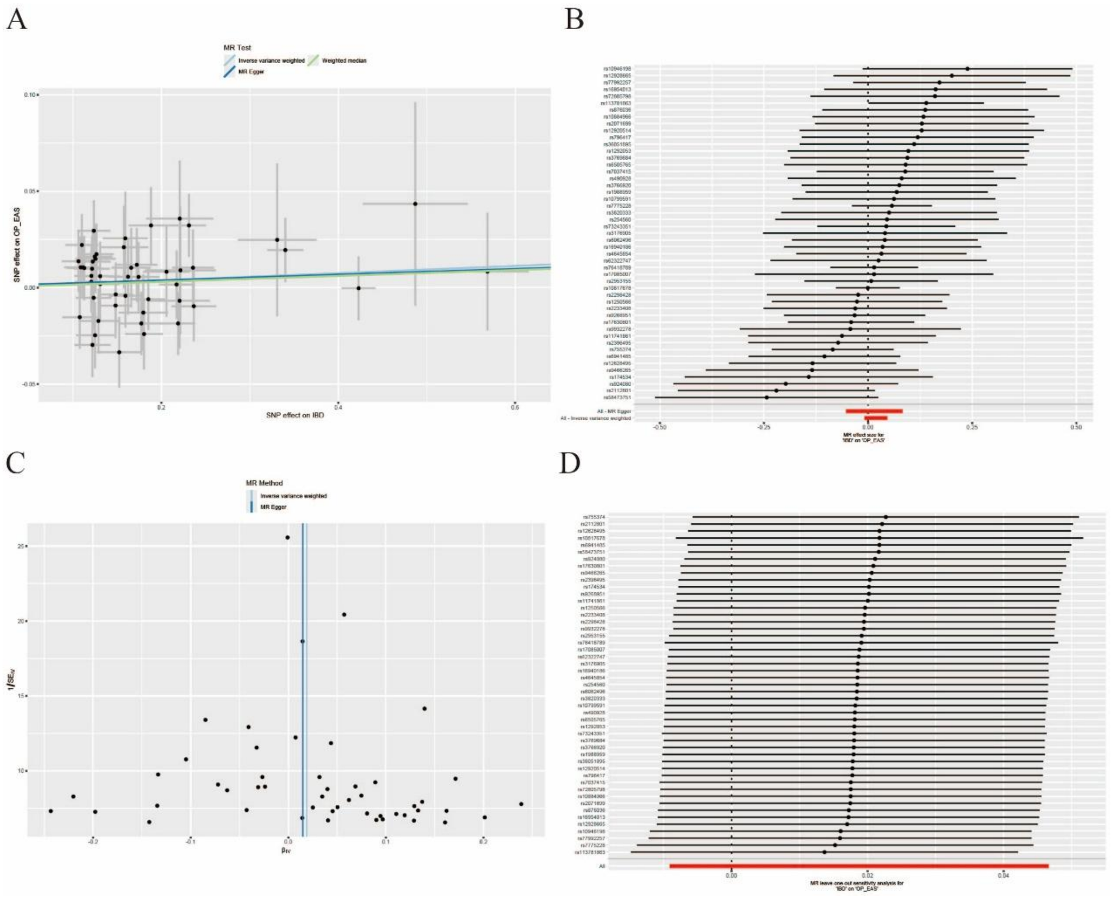

Figure S4 Scatterplot,funnel plot and leave-one-out analysis of relationship between IBD and OP in East Asian population.

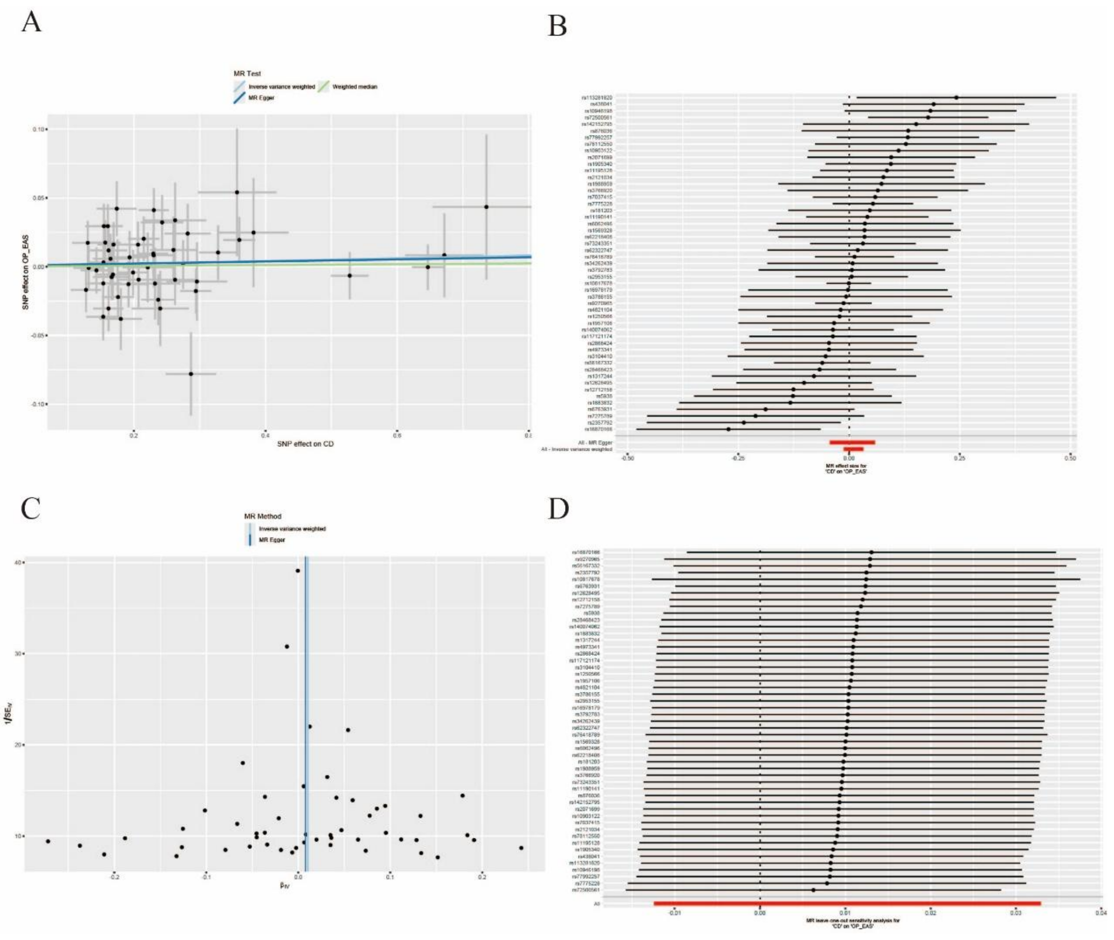

Figure S5 Scatterplot,funnel plot and leave-one-out analysis of relationship between CD and OP in East Asian population.

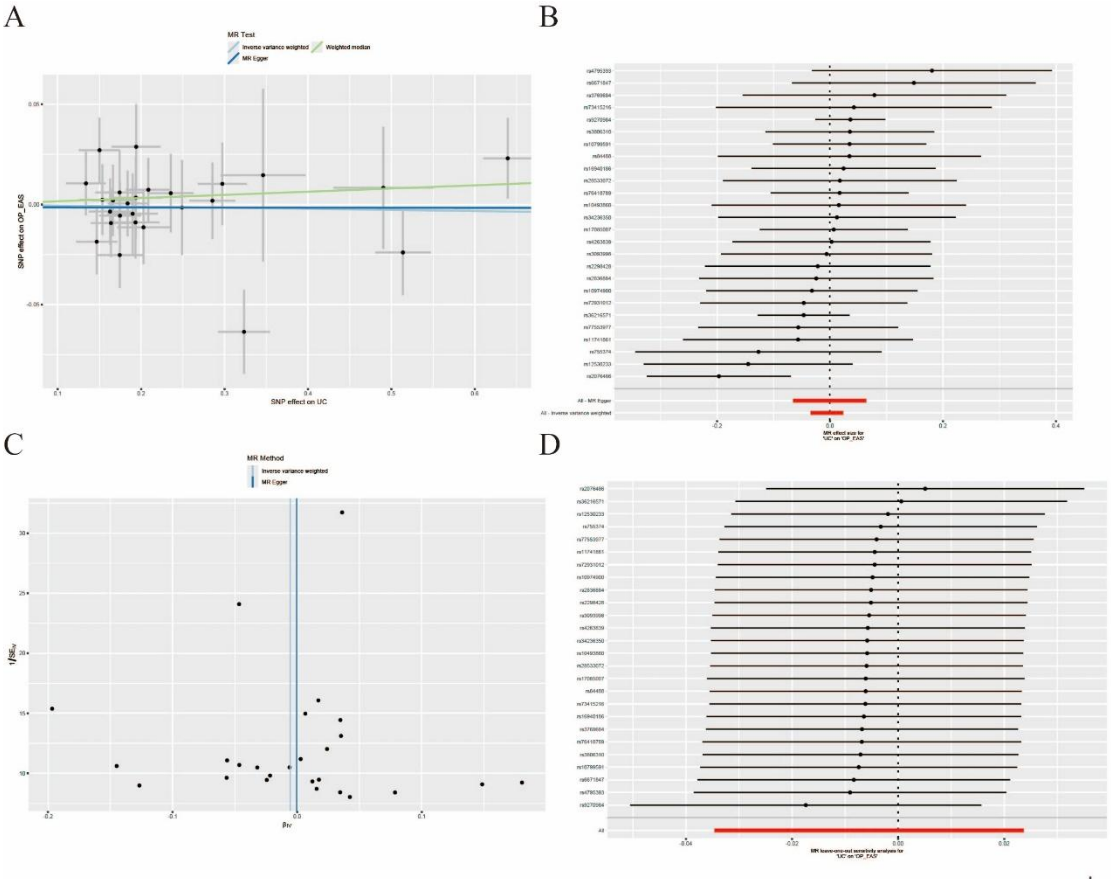

Figure S6 Scatterplot,funnel plot and leave-one-out analysis of relationship between UC and OP in East Asian population.

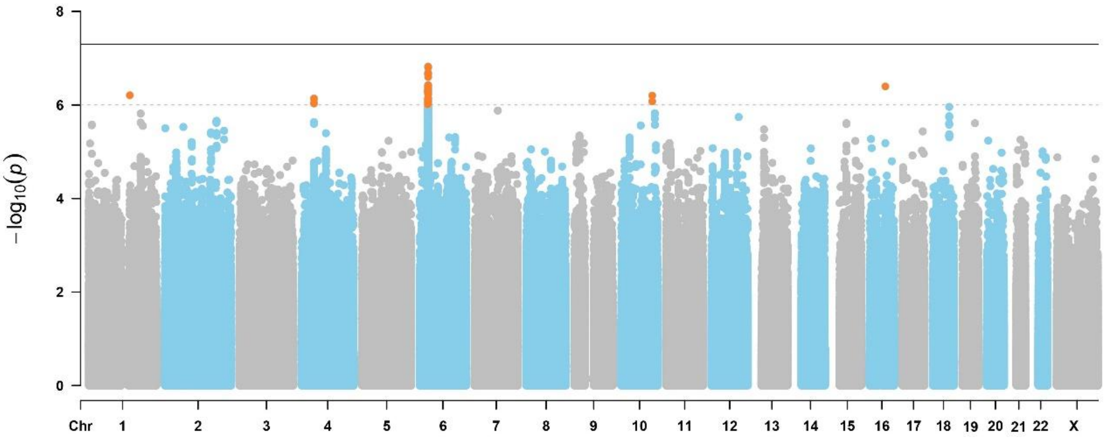

Figure S7 Manhattan Plot for Osteoporosis GWAS in the East Asian Population. Horizontal coordinates are chromosomes and vertical coordinates are -log10(p). The dashed line indicates p threshold of 1×10^{-6}, and the solid line indicates p threshold of 5×10^{-8}. Orange dots indicate SNPs $1\times10^{-6}<p<5\times10^{-8}$.

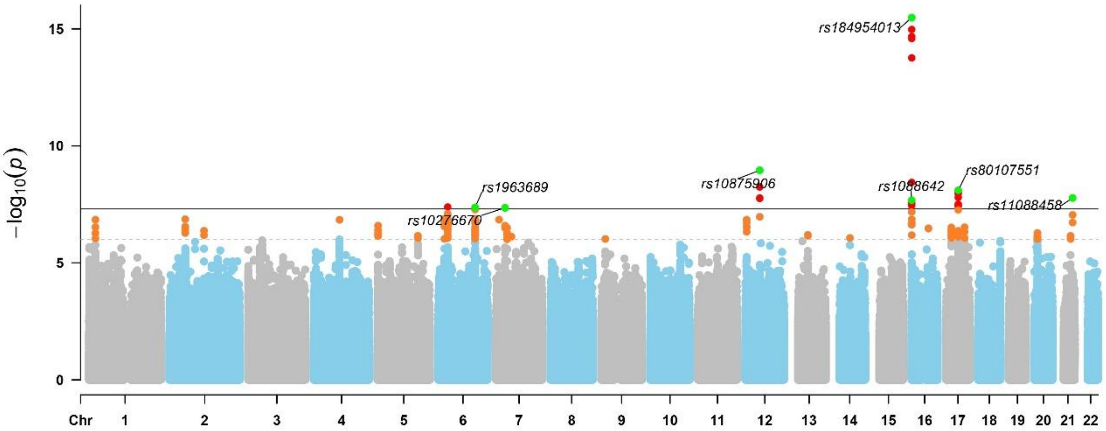

Figure S8 Manhattan Plot for Osteoporosis GWAS in the European Population. Horizontal coordinates are chromosomes and vertical coordinates are -log10(p). The dashed line indicates p threshold of 1×10^{-6}, and the solid line indicates p threshold of 5×10^{-8}. Orange dots indicate SNPs $1\times10^{-6}<p<5\times10^{-8}$.
